# Optimizing adipogenic cocktail composition to enhance beige adipogenesis and evaluate thermogenic potential in primary mouse subcutaneous fat cell cultures

**DOI:** 10.1038/s41366-025-01946-8

**Published:** 2025-11-24

**Authors:** Yujie Ma, Zijie Zhang, Erika Thalia Ramos, Elias Martin, Rahul Gunukula, Kara Sprabary, Heejin Jun

**Affiliations:** 1 Department of Nutritional Sciences, College of Health & Human Sciences, Texas Tech University, Lubbock, TX USA; 2 Department of Nutrition and Health Sciences, University of Nebraska-Lincoln, Lincoln, NE USA; 3 Honors College, Texas Tech University, Lubbock, TX USA

**Keywords:** Biological techniques, Cell biology

## Abstract

**Background:**

Subcutaneous white adipose tissue (WAT) is crucial for systemic metabolic homeostasis, with beige adipocytes in this depot contributing to energy metabolism through inducible thermogenesis. Differentiated adipocyte cultures derived from mouse inguinal WAT are a widely used system to study beige adipose biology and identify therapeutic targets, as they retain the genetic and epigenetic traits of native adipocytes while providing experimental flexibility. However, variability during the adipocyte induction and differentiation poses a challenge, impacting beige adipogenesis and experimental outcomes.

**Methods:**

This study conducted an unbiased analysis of four distinct adipogenic cocktails to evaluate their effects on beige adipogenesis in inguinal stromal vascular fraction cells from wild-type and genetically modified mice, as well as on the thermogenic activation of differentiated adipocytes.

**Results:**

Different combinations of adipogenic inducers, including dexamethasone, 3-isobutyl-1-methylxanthine, insulin, triiodothyronine, indomethacin, and rosiglitazone (Rosi), recruited beige adipocytes with varying levels of thermogenic characteristics. The peroxisome proliferator-activated receptor gamma agonist, Rosi, emerged as a key inducer, maximizing beige adipocyte biogenesis during the differentiation phase rather than the induction phase. However, Rosi-enhanced beige adipocyte differentiation exhibited limited thermogenic activation at the transcriptional level but not at the rapid signal transduction and real-time functional level in response to a β-adrenergic receptor agonist.

**Conclusions:**

These findings underscore the importance of optimizing adipogenic cocktails, as they significantly influence experimental outcomes. This study offers valuable guidance for selecting effective combinations of adipogenic inducers tailored to specific research objectives and relevant in vitro models of beige adipose biology.

## Introduction

Adipose tissue is a key endocrine organ composed of diverse cell types, including adipocytes, immune cells, endothelial cells, and stromal stem cells, that collectively regulate energy storage, heat generation, hormone secretion, and systemic energy homeostasis [1–4]. Depending on their distribution across various depots, adipose tissues contain three distinct adipocyte types—white, beige, and brown—each characterized by unique functions and regulatory mechanisms [5]. Understanding the unique roles and regulation of these adipocyte types is essential for elucidating adipose tissue function in both health and disease.

White adipocytes, located in subcutaneous and visceral white adipose tissue (WAT) in both rodents and humans, are unilocular cells with sparse mitochondria. These cells primarily function as energy storage units, and their expansion is associated with inflammation and obesity [6]. Brown adipocytes, predominantly found in the interscapular region of rodents within brown adipose tissue (BAT), originate from progenitors expressing somite markers, such as myogenic factor 5 and paired-box protein 3 [7, 8]. In humans, brown adipocytes are most abundant during infancy [9]. These multilocular cells are rich in mitochondria and uncoupling protein 1 (UCP1), a thermogenic marker in the inner mitochondrial membrane that drives non-shivering thermogenesis by dissipating energy into heat rather than ATP [9]. Another type of thermogenic fat cell, beige adipocytes, is highly inducible and predominantly found in subcutaneous WAT in both humans and rodents [10, 11]. Beige adipocytes emerge through a process known as “beiging” or “browning” in response to various stimuli, such as cold temperature [12]. In adult humans, functional thermogenic BAT, composed of brown and/or beige adipocytes, has been identified in regions such as the axillary, supraclavicular, cervical, paravertebral, and gluteofemoral areas [13]. The prevalence and distribution of BAT are influenced by factors including age, sex, body mass index, environmental temperature, and pharmaceutical interventions [14].

Both brown and beige adipocytes play crucial roles in energy metabolism beyond just heat production and maintaining body temperature. They contribute to systemic glucose and lipid homeostasis and enhance insulin sensitivity by acting as metabolic sinks for excess energy while secreting batokines [4]. These functions highlight their therapeutic potential for obesity, type 2 diabetes, and other cardiometabolic disorders. Recent studies also show that BAT activation can reduce glucose availability to tumors and slow their growth in both rodents and humans, paving the way for new health interventions leveraging these tissues [15]. Thus, enhancing the thermogenic capacity of adipose tissue may offer considerable health benefits. Beige adipocytes are particularly attractive due to their high inducibility in subcutaneous WAT, the most abundant adipose depot in humans, and their remarkable plasticity in response to genetic and external factors [16–19]. Understanding the autonomous biogenesis and regulatory mechanisms of beige adipocytes, as well as identifying small molecules that enhance their activity and abundance, is therefore of great interest.

The stromal vascular fraction (SVF) of rat and human adipose tissue was first recognized for its adipogenic potential in the 1970s, attributed to the presence of preadipocytes [20–22]. Accordingly, adipocytes differentiated from the SVF cells of murine subcutaneous inguinal WAT (IWAT) under adipogenic conditions are widely used as an in vitro model alongside in vivo animal study in beige adipose research. However, generating adipocytes from inguinal SVF cells poses challenges, including inconsistent or suboptimal differentiation, which can lead to variability in the molecular and functional characteristics of the resulting beige adipocytes and their responses to stimuli.

This study aims to evaluate various adipogenic cocktails for in vitro beige adipocyte differentiation from primary mouse IWAT SVF cells. It provides guidance for optimized protocols tailored to specific experimental objectives, including thermogenic activation for therapeutic target discovery and modeling beige adipose biology.

## Material and methods

### Animals

All experimental procedures involving animals were approved by the Institutional Animal Care and Use Committee at Texas Tech University (PRO22015-02). Wild-type C57BL/6J (Stock #000664), PR/SET domain 16 floxed (*Prdm16*^fl/fl^) (Stock #024992), Adiponectin (*Adipoq*)-Cre (Stock #028020), CByB6F1/J (Stock # 100009), C3D2F1/J (Stock #100004), and fibroblast growth factor 21 (*Fgf21*) knockout (KO) (Stock #033846) mice were obtained from Jackson Laboratory (Bar Harbor, MN, USA). Fat-specific *Prdm16* KO mice were produced by crossing *Prdm16*^fl/fl^ and *Adipoq*-Cre mice. UM-HET3 mice were generated by breeding CByB6F1/J females and C3D2F1/J males. Mice were maintained in a specific pathogen-free facility in a 12-h light/dark cycle (7 a.m.–7 p.m.) with free access to a standard chow diet and water.

### Primary mouse inguinal SVF isolation and culture

SVF cells from IWAT of 7–10-week-old male and female mice were isolated and cultured as previously reported with minor modification [23]. Briefly, IWAT was pooled, minced using surgical scissors, and digested in PBS (Millipore Sigma, #D8537, St. Louis, MO, USA) containing 1.5 U/mL collagenase D (Roche, #11088882001, Indianapolis, IN, USA), 2.4 U/mL dispase II (Roche, #4942078001, Indianapolis, IN, USA), and 10 mM CaCl_2_ at 37 °C with agitation (300 rpm). Digested tissues were suspended in wash medium [DMEM/F-12 GlutaMAX (Gibco, #10565018, Grand Island, NY, USA) supplemented with 10% fetal bovine serum (FBS) (MilliporeSigma, #F0926, St. Louis, MO, USA) and 1% penicillin-streptomycin], filtered through a 100-µm cell strainer (Corning, #431752, Tewksbury, MA, USA), and centrifuged at 2000 rpm for 3 min. The obtained cell pellet was suspended in wash medium, filtered with a 40-µm cell strainer (Corning, #431750, Tewksbury, MA, USA), and centrifuged as above. Isolated SVF cells were suspended in culture medium [DMEM/F-12 GlutaMAX supplemented with 10% FBS (MilliporeSigma, #F2442, Burlington, MA, USA) and 1% penicillin-streptomycin] and plated onto a 10 cm collagen-coated cell culture dish. Cells were grown at 37 °C with 5% CO_2_ and subcultured once with a split ratio of 1:2 or 1:3 when they reached 90% confluence. The same batch of FBS was used consistently for both cell isolation and culture throughout the study. The absence of mycoplasma contamination was routinely confirmed using the MycAway Plus-Color One-Step Mycoplasma Detection Kit (Yeasen Biotech, Gaithersburg, MD, USA).

### Induction and differentiation of primary mouse inguinal preadipocytes

The cultured primary mouse inguinal preadipocytes were seeded into 12-well collagen-coated plates at a cell density of approximately 12,500 cells per well. Confluent cells were induced and differentiated in the culture medium used for SVF cell culture following the four different recipes of the adipogenic cocktail provided in Tables 1 and S1. For induction, cells were stimulated for 2.5 days using the following agents from MilliporeSigma (St. Louis, MO, USA): dexamethasone (#D4902), 3-isobutyl-1-methylxanthine (IBMX) (#I7018), triiodothyronine (T3) (#T5516), insulin (#I5500), indomethacin (#I8250), or rosiglitazone (Rosi) (#R2408), refreshing the induction medium once. Induced SVF cells were maintained in a differentiation medium containing insulin, Rosi, or T3 for 4 days, refreshing the medium at the 2-day mark. Both induction and differentiation media were prepared fresh. At the end of the differentiation period, isoproterenol (Iso; 10 µM final concentration) was added for the designated experiments.

**Table 1:** Adipogenic cocktail recipes for differentiating primary mouse inguinal preadipocytes.

Recipe	Phase	Chemical	Working concentration
R1 [29–31]	Induction	Dexamethasone	1 µM
		IBMX	0.5 mM
		Insulin	850 nM
	Differentiation	Insulin	850 nM
R2 [33, 34, 36]	Induction	Dexamethasone	5 µM
		IBMX	0.5 mM
		Insulin	850 nM
		T3	1 nM
		Indomethacin	125 nM
	Differentiation	Insulin	850 nM
		T3	1 nM
R3 [17, 30, 31, 37]	Induction	Dexamethasone	5 µM
		IBMX	0.5 mM
		Insulin	85 nM
		Rosi	0.5 µM
	Differentiation	Insulin	85 nM
		Rosi	0.5 µM
R4 [38–41]	Induction	Dexamethasone	5 µM
		IBMX	0.5 mM
		Insulin	850 nM
		T3	1 nM
		Indomethacin	125 nM
		Rosi	0.5 µM
	Differentiation	Insulin	850 nM
		T3	1 nM
		Rosi	0.5 µM

### Oil Red O staining

Mature adipocytes differentiated from mouse inguinal preadipocytes in a 12-well plate were gently washed twice with PBS and fixed with 10% buffered formalin phosphate for 1 h at room temperature. Fixed cells were rinsed twice with filtered water, incubated with 60% isopropanol for 1 min and left dry at room temperature. Dried adipocytes were stained at a 3:2 ratio of Oil Red O solution (MilliporeSigma, #O1391, St. Louis, MO, USA) in filtered water at room temperature for 10 min and washed with filtered water until the water coloration was clear. Oil Red O-stained adipocytes were imaged using the BioTek Cytation 5 (Agilent, Santa Clara, CA, USA) imager. The Oil Red O dye extracted from stained lipid droplets with isopropanol was quantified by measuring absorbance at 492 nm using the BioTek Synergy H1 Multimode Reader (Agilent, Santa Clara, CA, USA) [24].

### 4,4-difluoro-4-bora-3a,4a-diaza-s-indacene (BODIPY) 493/503 staining

Primary mouse IWAT preadipocytes were seeded into collagen-coated coverslips and differentiated to mature adipocytes. Fully differentiated adipocytes were washed twice with PBS and fixed with 4% buffered formalin phosphate for 10 min. After washing twice with PBS, fixed cells were incubated with 2 µM of BODIPY 493/503 (Invitrogen, #D3922, Carlsbad, CA, USA) in PBS and 1 µg/mL DAPI (Thermo Scientific, #62247, Rockford, IL, USA) to stain neutral lipids and nuclei, respectively, for 15 min at room temperature in the dark. Stained adipocytes were washed with PBS twice and mounted using ProLong Gold Antifade Mountant (Invitrogen, #P10144, Eugene, OR, USA) and allowed to cure for 24 h. Images of BODIPY and DAPI-stained adipocytes were taken using the EVOS FL Inverted Fluorescence Microscope (Invitrogen, Carlsbad, CA, USA).

### Quantitative real-time PCR (qPCR)

Total RNA was extracted using TRI reagent (MilliporeSigma, #T9424, Burlington, MA, USA). cDNA was synthesized using 1–2 µg of RNA according to the manufacturer’s protocol for M-MLV Reverse Transcriptase (Life Technologies, #28025021, Carlsbad, CA, USA). qPCR was performed using SYBR Green (Life Technologies, #A25742, Carlsbad, CA, USA) in the QuantStudio 6 384-well system (Thermo Scientific, Rockford, IL, USA). Relative gene expression levels were calculated by the 2^−ΔΔ*Ct*^ method and normalized to TATA box-binding protein (*Tbp*), which serves as a stable reference gene during adipogenesis and drug treatments [25, 26]. Primers sequences are listed in Table S2.

### Western blotting

Fully differentiated adipocytes were washed twice with cold PBS and lysed in cold RIPA buffer (1% NP-40, 0.5% sodium deoxycholate, 150 mM NaCl, 50 mM Tris-Cl, pH 7.5, 0.1% SDS) supplemented with protease (Roche, #11836153001, Indianapolis, IN, USA) and phosphatase (Thermo Scientific, #J61022AA, Carlsbad, CA, USA) inhibitor cocktails. Equal amounts of protein were loaded and separated onto SDS-PAGE and transferred onto nitrocellulose membranes. The membranes were incubated with primary antibodies against phospho-PKA substrate^S/T^ (1:1000, Cell Signaling, #9621S, Danvers, MA, USA), phospho-HSL^S563^ (1:2000, Cell Signaling, #4139S, Danvers, MA, USA), UCP1 (1:3000, Abcam, #ab10983, Cambridge, MA, USA), Total OXPHOS Rodent WB Antibody Cocktail (1:2000, Abcam, #ab110413, Cambridge, MA, USA), PPARG (1:2000, Cell Signaling, #2435S, Danvers, MA, USA), HSP90 (1:5000, Cell Signaling, #4874S, Danvers, MA, USA) or β-actin (1:5000, Cell Signaling, #8457S, Danvers, MA, USA). Proteins were visualized using enhanced chemiluminescence substrate reagents (Thermo Scientific, #32106, Carlsbad, CA, USA; Bio-Rad, #1705062, Hercules, CA, USA) and ChemiDoc system (Bio-Rad, Hercules, CA, USA). Band intensities were quantified using ImageJ software (NIH, Bethesda, MD, USA).

### Oxygen consumption rate (OCR)

Primary mouse IWAT preadipocytes were seeded and fully differentiated in a 96-well plate. The OCR of mature adipocytes was measured in culture medium for 2 h using the Extracellular Oxygen Consumption Assay Kit (Abcam, #ab197243, Cambridge, MA, USA) according to the manufacturer’s instructions. Data were expressed as changes relative to the control average for comparative analysis.

### Statistical analysis

GraphPad Prism 10 (GraphPad Software, Inc., La Jolla, CA, USA) was used for generating figures and statistical analysis. Data are presented as mean ± standard error of the mean (SEM). Sample sizes were determined on the basis of preliminary data and prior publications. Results were replicated in at least two independent experiments. Normality was assessed with the Shapiro–Wilk test for 3 ≤ *n* ≤ 7 and the D’Agostino–Pearson omnibus test for *n* ≥ 8. Two-group comparisons were performed using a two-tailed Student’s *t* test for normally distributed data and Mann–Whitney *U* test otherwise. For comparisons among four groups with one factor, parametric analyses used one-way ANOVA with Tukey’s post hoc test (equal variances) or Welch’s ANOVA with Dunnett’s T3 (unequal variances). For nonparametric data, Kruskal–Wallis test followed by Dunn’s post hoc test was used. A *p* value < 0.05 was considered statistically significant.

## Results

### Beige adipogenesis with different adipogenic cocktails

**Fig. 1:**
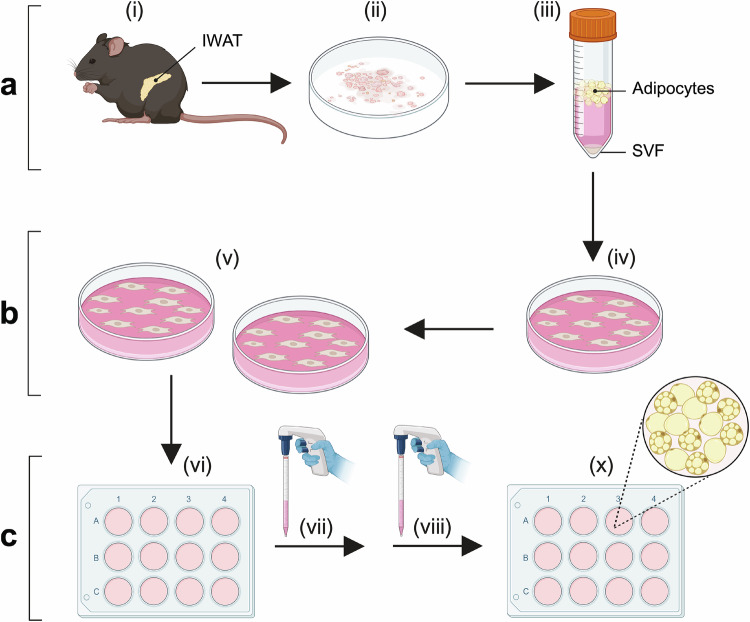
Flow scheme illustrating the generation of beige adipocytes from mouse primary subcutaneous white adipose stromal vascular fraction (SVF) through a standard three-step process. **a** Inguinal white adipose tissues (IWAT) were collected from mice (i), pooled (ii), and enzymatically digested (iii) to isolate SVF via centrifugation-based precipitation. **b** The isolated IWAT SVF cells were plated (iv) and subcultured in cell culture dishes to allow preadipocyte proliferation (v). **c** IWAT preadipocytes were seeded into well plates (vi), cultured to confluence, and induced to differentiate (vii and viii) into mature adipocytes (x) using four distinct adipogenic cocktails (R1, R2, R3, and R4) tested in this study. Created with BioRender.com.

Beige adipocytes predominantly arise in subcutaneous WAT, with IWAT being the largest subcutaneous adipose depot in mice [10, 27]. In this study, mouse beige adipocytes were generated through a three-step process: adipose SVF isolation, culture, and differentiation (Fig. 1) [23]. IWAT from 5–6 mice were dissected, pooled, minced, and enzymatically digested with collagenase D and dispase II, followed by centrifugation to precipitate SVF cells. The isolated cells were cultured and expanded on culture dishes. Once seeded and grown to confluence in 12-well plates, the SVF cells were differentiated into mature adipocytes (Fig. 1).

Adipogenesis from preadipocytes is achieved through two sequential phases: the induction phase, which promotes growth arrest and commitment to the adipocyte lineage, and the differentiation phase, which supports the morphological and metabolic maturation of adipocytes [28]. This process relies on exogenous adipogenic inducers. In addition to FBS, compounds such as IBMX, dexamethasone, insulin, Rosi, T3, and indomethacin are commonly included in adipogenic cocktails to drive the complex gene expression program required for adipogenesis. The combinations of these compounds in adipogenic cocktails for beige adipocyte biogenesis from murine subcutaneous preadipocytes vary considerably across studies.

To evaluate how this variability affects beige adipogenic efficacy, we conducted an unbiased comparison of four adipogenic cocktails previously used in studies (recipes R1, R2, R3, and R4) in cultured IWAT SVF cells from wild-type C57BL/6J mice (Tables 1 and S1). The R1 adipogenic cocktail represents the minimal combination of chemicals required for complete adipocyte differentiation. It consists of dexamethasone, a synthetic glucocorticoid agonist; IBMX, a phosphodiesterase inhibitor that increases intracellular cyclic adenosine monophosphate (cAMP) and activates protein kinase A (PKA); and insulin, which facilitates glucose uptake and lipogenesis [29–31]. Commonly referred to as MDI [Methylisobutylxanthine (IBMX), Dexamethasone, and Insulin], this cocktail is widely accepted as the standard method for inducing differentiation in the 3T3-L1 murine preadipocyte cell line but is not commonly used for promoting beige adipogenesis in murine primary inguinal preadipocytes [28, 32]. R2 included dexamethasone, IBMX, insulin, indomethacin—an activator of peroxisome proliferator-activated receptor gamma (PPARγ)—and T3, which promotes adipogenic and lipogenic gene transcription via thyroid hormone receptor α [33–36]. R3 consisted of IBMX, dexamethasone, insulin, and Rosi, a potent PPARγ agonist [17, 30, 31, 37]. Finally, R4 incorporated all of the adipogenic inducers: IBMX, dexamethasone, insulin, indomethacin, T3, and Rosi [38–41].

**Fig. 2:**
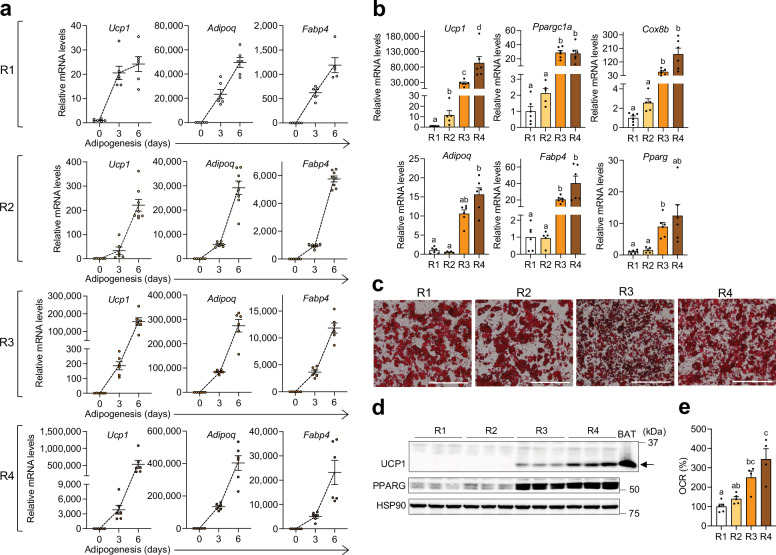
Beige adipogenesis in primary inguinal preadipocytes from wild-type C57BL/6J mice under four distinct adipogenic cocktails. **a** qPCR analysis of thermogenic (*Ucp1*) and adipogenic (*Adipoq* and *Fabp4*) markers at 0, 3, and 6.5 (endpoint) days of differentiation using adipogenic recipes R1 (*n* = 5–6/group), R2 (*n* = 6–8/group), R3 (*n* = 6/group), and R4 (*n* = 6/group) in primary inguinal preadipocyte cultures. **b** qPCR analysis of thermogenic (*Ucp1*, *Ppargc1a*, and *Cox8b*) and adipogenic (*Adipoq*, *Fabp4*, and *Pparg*) gene expression in fully differentiated mature inguinal adipocytes derived with different adipogenic recipes (R1, *n* = 6; R2, *n* = 5; R3, *n* = 6; R4, *n* = 6). **c** Representative images of Oil Red O-stained mature inguinal adipocytes differentiated using the four adipogenic cocktails. Scale bar: 200 µm. **d** Immunoblot analysis of UCP1 and PPARG in mature adipocytes differentiated with the four adipogenic recipes, with HSP90 used as a loading control (*n* = 3/group). Brown adipose tissue (BAT) was included as a positive control for UCP1, indicated by the arrow. **e** Oxygen consumption rate (OCR) in mature adipocytes differentiated with different adipogenic recipes (R1 and R2, *n* = 5; R3 and R4, *n* = 4). Data are presented as mean ± SEM. Four-group comparisons were assessed by one-way ANOVA with Tukey’s post hoc test, Welch’s ANOVA with Dunnett’s T3, or Kruskal–Wallis test with Dunn’s post hoc test. Different letters indicate statistically significant differences at *p* < 0.05.

All four recipes significantly induced *Ucp1*, a key thermogenic fat marker, alongside adipogenic genes such as adiponectin (*Adipoq*) and fatty acid-binding protein 4 (*Fabp4*) during beige adipogenesis of primary inguinal preadipocytes (Fig. 2a). However, recipes R3 and R4 exhibited greater adipogenic efficacy compared to R1 and R2, as indicated by higher mRNA levels of *Adipoq*, *Fabp4*, and *Pparg* (Fig. 2b). Consistently, Oil Red O staining confirmed differentiation across all recipes, with R3 and R4 showing greater adipogenesis (Figs. 2c and S1a). Additionally, the expression of thermogenic markers, including peroxisome proliferator-activated receptor gamma coactivator 1-alpha (*Ppargc1a*), cytochrome c oxidase subunit 8B (*Cox8b*), and *Ucp1*, was significantly higher in R3 and R4 than in R1 and R2 (Fig. 2b). Among the four adipogenic cocktails, R4 demonstrated the highest overall adipogenic and thermogenic effectiveness (Fig. 2b). R1 and R2 yielded comparable adipogenesis, but R2 showed modestly higher thermogenic transcription than R1. Despite robust *Ucp1* mRNA induction across all cocktails, UCP1 protein was undetectable in R1- and R2-derived adipocytes and readily detected in R3 and R4, with the strongest signal in R4 (Figs. 2d and S1b). Consistent with mitochondrial enrichment as a hallmark of thermogenic adipocytes, R3 and R4 showed higher expression of mitochondrial respiratory chain complex proteins than R1 and R2 (Fig. S1c) [4]. Functional readouts based on OCR supported the molecular data but revealed more modest differences among recipes (Fig. 2e). These findings highlight that varying combinations of adipogenic inducers result in distinct adipogenic and thermogenic outcomes.

### The critical role of rosiglitazone in maximizing beige adipogenesis

We aimed to identify the adipogenic inducer primarily responsible for the observed differences in thermogenic efficacy. Recipes R1 and R3 share a similar chemical composition, with the key distinction being the inclusion of Rosi in R3. Similarly, recipes R2 and R4 differ only by the addition of Rosi in R4. Rosi, a synthetic PPARγ agonist belonging to the thiazolidinedione (TZD) class, was initially developed as an insulin sensitizer for type 2 diabetes. Identified in the mid-1990s, PPARγ is adipose-enriched and essential for adipogenesis [42]. Accordingly, PPARγ agonists are used to promote adipogenesis in cell culture systems. Furthermore, chronic treatment with Rosi during adipogenesis of murine primary inguinal preadipocytes and mesenchymal stromal cells has been shown to robustly activate the thermogenic molecular program [36, 43]. Consistent with these observations, our data indicate that Rosi significantly enhances beige adipogenesis when included as part of the adipogenic cocktail.

**Fig. 3:**
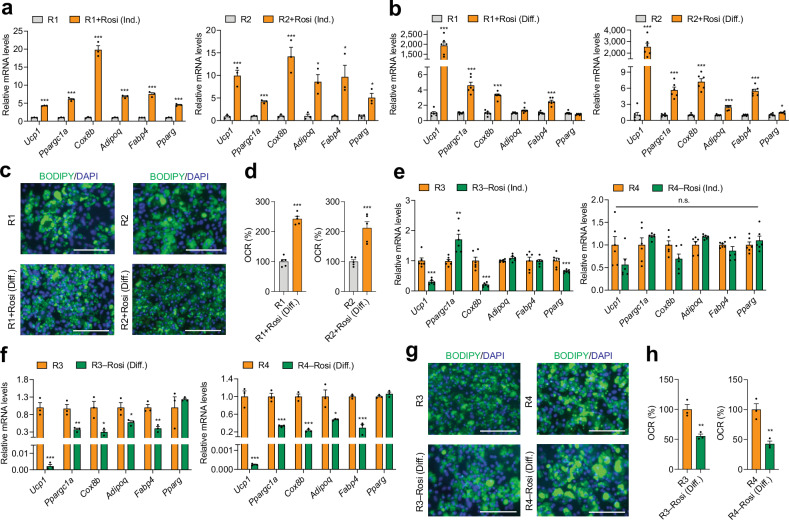
The critical role of rosiglitazone (Rosi) in enhancing beige adipogenesis in primary inguinal preadipocytes from wild-type C57BL/6J mice. **a**, **b** qPCR analysis of thermogenic (*Ucp1*, *Ppargc1a*, and *Cox8b*) and adipogenic (*Adipoq*, *Fabp4*, and *Pparg*) gene expression in inguinal adipocytes differentiated under adipogenic recipes R1 and R2, with or without Rosi supplementation during the **a** induction phase (*n* = 3/group), or **b** differentiation phase (*n* = 6/group). **c** Representative images of inguinal adipocytes stained with BODIPY (green, neutral lipids) and DAPI (blue, nuclei) after differentiation using adipogenic cocktails R1 and R2 with or without Rosi supplementation during the differentiation phase. Scale bar: 150 µm. **d** Oxygen consumption rate (OCR) in mature adipocytes differentiated with adipogenic recipes R1 and R2 with or without Rosi supplementation during the differentiation phase (*n* = 5/group). qPCR analysis of thermogenic and adipogenic gene expression in inguinal adipocytes differentiated under adipogenic recipes R3 and R4, as well as R3 and R4 without Rosi, during the **e** induction phase (*n* = 6/group), or **f** differentiation phase (*n* = 3/group). **g** Representative images of BODIPY and DAPI-stained inguinal adipocytes differentiated using adipogenic cocktails R3 and R4, as well as R3 and R4 without Rosi, during the differentiation phase. Scale bar: 150 µm. **h** OCR in mature adipocytes differentiated with adipogenic recipes R3 and R4 with Rosi either retained or removed during the differentiation phase (*n* = 3/group). Data are presented as mean ± SEM. Two-group differences were assessed using two-tailed Student’s *t* test or Mann–Whitney *U* test. *p < 0.05; **p < 0.01; ***p < 0.001; n.s., non-significant difference.

To pinpoint the specific phases of in vitro beige adipogenesis—induction or differentiation—where Rosi exerts its most critical effects, we conducted further investigations. We supplemented Rosi into recipes R1 and R2, which lack Rosi, during either the induction or differentiation phase of adipogenesis in cultured IWAT preadipocytes from C57BL/6J mice. Supplementing Rosi during either phase enhanced beige adipogenesis overall after full differentiation, as indicated by increased mRNA expression of thermogenic (*Ucp1*, *Ppargc1a*, and *Cox8b*) and general adipogenic (*Adipoq*, *Fabp4*, and *Pparg)* markers (Fig. 3a, b). Notably, thermogenic enhancement was significantly more pronounced when Rosi was added during the differentiation phase, rather than the induction phase, as evidenced by a marked elevation in *Ucp1* expression (Fig. 3a, b). Morphological assessment using BODIPY staining revealed a noticeable increase in multilocular lipid droplets in mature adipocytes, characteristic of thermogenic adipocytes, when Rosi was present during the differentiation phase in recipes R1 or R2 (Fig. 3c). OCR was also higher with differentiation-phase Rosi, indicating greater thermogenic capacity at the functional level (Fig. 3d). Conversely, removing Rosi from R3 and R4 during the differentiation phase led to a greater suppression of adipogenic and thermogenic gene programs than its removal during the induction phase and yielded undetectable UCP1, as observed in fully differentiated adipocytes (Figs. 3e, f and S2). BODIPY staining revealed larger lipid droplets in mature adipocytes developed without Rosi during the differentiation phase (Fig. 3g). Consistently, OCR-based functional assessment demonstrated markedly reduced respiration in the absence of Rosi (Fig. 3h). These findings underscore Rosi’s critical role in enhancing beige adipogenesis by promoting both adipogenic and thermogenic gene programs, with its impact being particularly significant during the differentiation phase.

### Thermogenic activation in mature inguinal adipocytes differentiated with different adipogenic cocktails

Differentiated murine subcutaneous adipocytes are widely used as an in vitro platform to screen and evaluate the thermogenic activation potential of small molecules. To investigate how different adipogenic recipes influence the thermogenic characteristics of beige adipocytes in response to external stimuli, we examined β-adrenergic receptor (β-AR)-mediated responses in mature adipocytes differentiated with the four cocktails. β-ARs, which include three subtypes—β1, β2, and β3—play essential roles in regulating thermogenesis and associated lipolysis, with β3 being the predominant subtype expressed in thermogenic adipocytes [44].

**Fig. 4:**
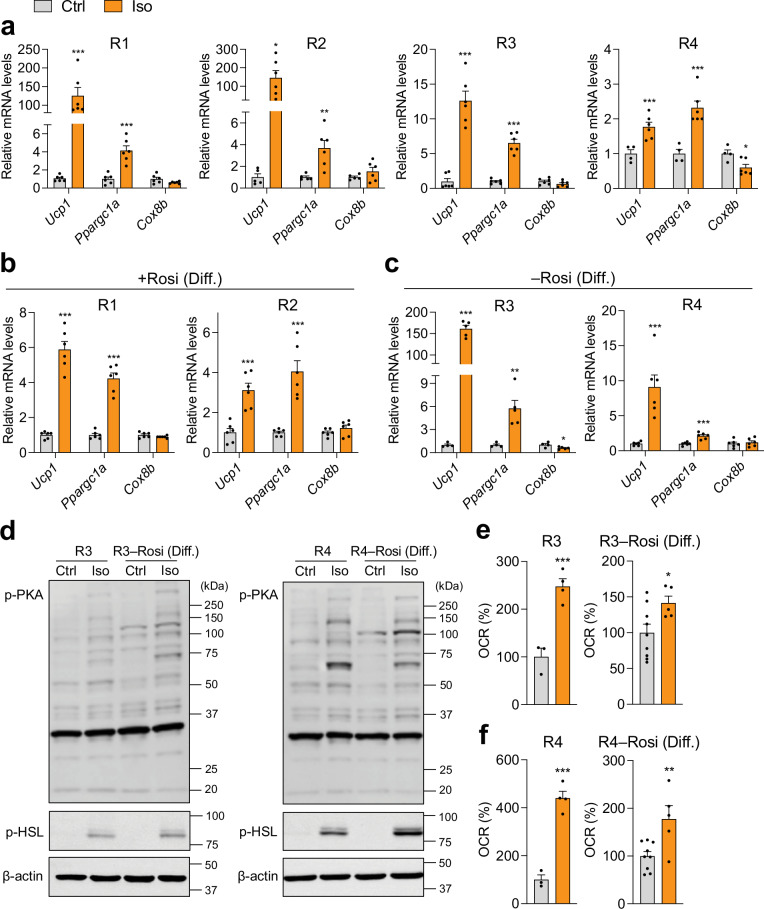
β-adrenergic signaling-mediated thermogenic activation in mature adipocytes differentiated from primary inguinal preadipocytes of wild-type C57BL/6J mice under distinct adipogenic cocktails. **a** qPCR analysis of thermogenic markers following vehicle (Ctrl) or 10 µM Iso treatment for 4 h in inguinal adipocytes differentiated with four different adipogenic cocktails: R1 (*n* = 6/group), R2 (*n* = 5–6/group), R3 (*n* = 6/group), and R4 (*n* = 4–6/group), after treatment with control (Ctrl) or 10 µM isoproterenol (Iso) for 4 h. qPCR analysis of thermogenic gene expression in mature inguinal adipocytes differentiated under adipogenic recipes: **b** R1 and R2 supplemented with rosiglitazone (Rosi) at the differentiation phase (*n* = 6/group), or **c** R3 and R4 without Rosi during the differentiation phase (R3, *n* = 4–5/group; R4, *n* = 6/group). **d** Immunoblot analysis of phosphorylated PKA and HSL protein levels in inguinal adipocytes differentiated with adipogenic cocktails R3 and R4, with or without Rosi, following treatment with Ctrl or 10 µM Iso for 15 min. β-actin was used as a reference protein. Oxygen consumption rate (OCR) changes in response to 10 µM Iso in mature adipocytes differentiated with **e** R3 and **f** R4, with Rosi either retained or removed during the differentiation phase (R3 and R4, *n* = 3–4/group; R3 and R4 without Rosi, *n* = 5–9/group). Data are presented as mean ± SEM. Two-group differences were assessed using two-tailed Student’s *t* test or Mann–Whitney *U* test. *p < 0.05; **p < 0.01; ***p < 0.001.

We treated fully differentiated inguinal mature adipocytes from C57BL/6J mice with isoproterenol (Iso), a pan β-AR agonist, for 4 h and evaluated thermogenic gene activation. Adipocytes differentiated using recipes R1 and R2 exhibited greater induction of thermogenic genes (*Ucp1* and *Ppargc1a*) in response to Iso compared to those differentiated under R3 and R4 (Fig. 4a). This effect was likely due to the lower basal thermogenic activity in adipocytes differentiated with R1 and R2, which lacked Rosi. In contrast, adipocytes differentiated with recipes R3 and R4, which included Rosi, exhibited high or maximized basal thermogenic activity, thereby limiting their capacity for further thermogenic activation in response to Iso (Figs. 2c and 4a). When Rosi was added during the differentiation phase in recipes R1 and R2 to enhance thermogenic adipogenesis, Iso-induced thermogenic gene induction was less pronounced compared to the original recipes without Rosi (Fig. 4b). Conversely, recipes R3 and R4, modified to exclude Rosi during the differentiation phase and suppress basal thermogenic activity, showed improved thermogenic gene induction in response to Iso (Fig. 4c). It is of note that short-term activation of thermogenic adipocytes via β-AR signaling was insufficient to upregulate the mitochondrial gene *Cox8b* (Fig. 4a–c).

The functional consequence of β-AR activation involves elevated levels of the second messenger cAMP in thermogenic adipocytes, which leads to the phosphorylation of PKA and the key lipolysis protein, hormone-sensitive lipase (HSL). Iso-induced thermogenic activation, measured by PKA and HSL phosphorylation, was significant in mature beige adipocytes differentiated using recipes R3 and R4, irrespective of Rosi’s presence during the differentiation phase (Fig. 4d). We also quantified the real-time response to Iso using the OCR assay. R3- and R4-derived mature adipocytes exhibited significantly larger Iso-induced OCR increases than cells generated without Rosi during the differentiation phase (Fig. 4e, f). These data indicate that potent cocktails such as R3 and R4 prime cells for rapid thermogenic activation with minimal reliance on slower transcriptional remodeling. Accordingly, integrating acute signaling and real-time functional readouts with gene expression analyses enables a more comprehensive, less cocktail-dependent assessment.

### Plasticity of beige adipogenesis with different adipogenic cocktails

One of the key characteristics of beige adipogenesis is its remarkable plasticity and adaptability, resulting in the generation of diverse subtypes of beige adipocytes with alternative thermogenic mechanisms in subcutaneous WAT, even under genetic modifications. In the absence of UCP1, beige adipogenesis can still occur, producing noncanonical beige adipocytes that generate heat through ATP hydrolysis via calcium cycling mediated by sarcoplasmic/endoplasmic reticulum calcium ATPase 2B and through creatine phosphorylation facilitated by mitochondrial creatine kinases [17, 45]. Additionally, β-less mice, which lack all three β-AR subtypes (β1, β2, and β3), can recruit glycolytic beige adipocytes derived from myogenic progenitors via GA-binding protein alpha [16]. Therefore, we evaluated the effects of four different adipogenic cocktails on beige adipogenesis in primary mouse inguinal preadipocytes lacking key thermogenic regulators, such as fibroblast growth factor 21 (*Fgf21*) and PR/SET domain 16 (*Prdm16*).

**Fig. 5:**
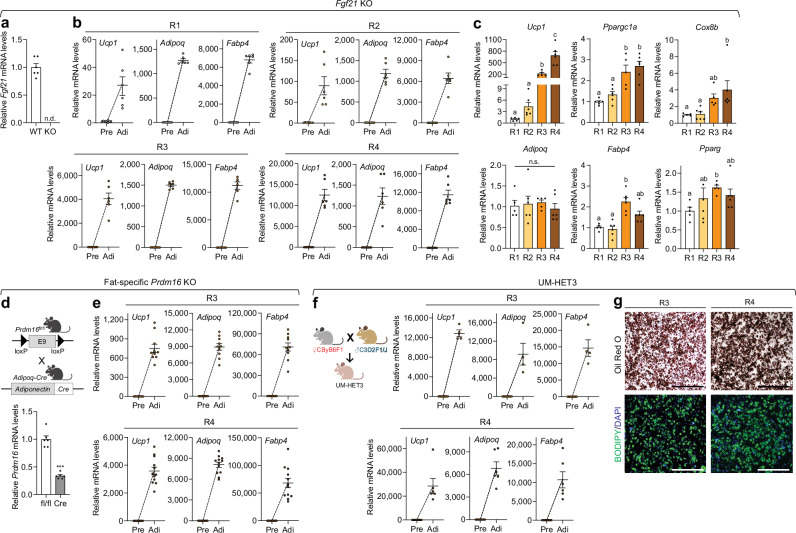
Beige adipogenesis with different adipogenic cocktails in primary inguinal preadipocytes from genetically modified and diverse mouse models. **a**–**c** Analysis in whole-body *Fgf21* KO mice. **a** qPCR analysis of *Fgf21* mRNA levels in inguinal white adipose tissue (IWAT) from wild-type (WT) control and *Fgf21* KO mice (*n* = 6/genotype). **b** qPCR analysis of thermogenic (*Ucp1*) and adipogenic markers (*Adipoq* and *Fabp4*) in inguinal preadipocytes and mature adipocytes differentiated using adipogenic recipes R1, R2, R3, and R4 (*n* = 6–8/group). **c** qPCR analysis of thermogenic (*Ucp1*, *Ppargc1a*, and *Cox8b*) and adipogenic (*Adipoq*, *Fabp4*, and *Pparg*) gene expression in fully differentiated inguinal mature adipocytes using different adipogenic recipes (R1, *n* = 5–6; R2, *n* = 6; R3, *n* = 5–6; R4, *n* = 6). **d**, **e** Analysis in fat-specific *Prdm16* KO mice. **d** Top: Schematic representation of fat-specific *Prdm16* KO generation by crossing *Prdm16*^fl/fl^ and *Adipoq*-Cre mice (created with BioRender.com). Bottom: qPCR analysis of *Prdm16* gene expression in IWAT from floxed control and KO mice (*n* = 6/genotype). **e** qPCR analysis of thermogenic and adipogenic markers in inguinal preadipocytes and mature adipocytes differentiated using adipogenic recipes R3 (*n* = 8–10/group) and R4 (*n* = 8–12/group). **f**, **g** Analysis in UM-HET3 mice. **f** Left: Schematic of UM-HET3 mouse generation via a four-way cross, achieved by mating CByB6F1/J hybrid females with C3D2F1/J hybrid males (created with BioRender.com). Right: qPCR analysis of thermogenic and adipogenic markers in inguinal preadipocytes and mature adipocytes differentiated using adipogenic recipes R3 (*n* = 4–8/group) and R4 (*n* = 6–8/group). **g** Representative images of (top) Oil Red O and (bottom) BODIPY and DAPI-stained mature inguinal adipocytes differentiated using adipogenic recipes R3 and R4. Scale bar: 500 µm. Data are presented as mean ± SEM. Four-group comparisons were assessed by one-way ANOVA with Tukey’s post hoc test, Welch’s ANOVA with Dunnett’s T3, or Kruskal–Wallis test with Dunn’s post hoc test. Different letters indicate statistically significant differences at *p* < 0.05. n.s., not significant.

FGF21 is one of the most well-known batokines, with its secretion increasing in response to cold exposure or β3-AR stimulation. It functions as an autocrine factor to promote thermogenesis in beige adipocytes [46, 47]. Consistent with observations in primary IWAT SVF cells from wild-type mice (Fig. 2a), those from *Fgf21* KO mice were also capable of differentiating into beige adipocytes under all four adipogenic cocktails (Fig. 5a, b). Interestingly, while mature adipocytes differentiated from *Fgf21* KO IWAT preadipocytes using recipes R3 and R4 exhibited a higher thermogenic gene program compared to those differentiated with R1 and R2, the inductions were less pronounced than those in wild-type cells (Figs. 2b and 5c). UCP1 protein was undetectable even in R3- and R4-derived adipocytes, consistent with reduced beige adipocyte abundance and/or activity due to disruption of key regulatory pathways relative to wild-type (Fig. S3a) [46, 48]. However, a significant rise in mitochondrial respiratory complex proteins confirmed recruitment of thermogenic beige adipocytes (Fig. S3a). Unlike wild-type adipocytes, *Fgf21* KO adipocytes showed comparable overall adipogenic gene expression levels across all tested adipogenic cocktails (Figs. 2b and 5c). Our findings support that beige adipocytes with *Fgf21*-independent thermogenic mechanisms can be recruited, highlighting the potential for exploring alternative thermogenic pathways. The influence of cocktail formulation on adipogenesis was less pronounced in inguinal preadipocytes lacking *Fgf21* than in wild-type cells. We further validated the emergence of beige adipocytes using recipes R3 and R4 in a separate set of primary IWAT SVF cells from mice with a defective thermogenic program caused by adipocyte-specific loss of *Prdm16*, a critical cell-autonomous activator of beige adipocyte biogenesis (Figs. 5d, e and S3b) [48].

Throughout this study, primary IWAT SVF cells tested with the adipogenic cocktails were derived from mice with a C57BL/6J genetic background. To evaluate the broader applicability of our findings, we employed UM-HET3 mice as a model for human genetic diversity. These mice, generated by crossing CByB6F1/J hybrid females with C3D2F1/J hybrid males, possess a genetically heterogeneous background derived from four widely used wild-type strains: C57BL/6J, BALB/cByJ, C3H/HeJ, and DBA/2J (Fig. 5f) [49]. Recipes R3 and R4 effectively induced beige adipocyte biogenesis at both transcriptional and morphological levels, as demonstrated by increased thermogenic gene expression and lipid droplet formation visualized through BODIPY staining in differentiated mature inguinal adipocytes from UM-HET3 mice (Fig. 5f, g). These findings indicate that the adipogenic cocktails tested in this study can successfully induce beige adipogenesis, with R3 and R4 emerging as the most effective for enhancing thermogenesis, even in models with impaired thermogenic capacity and a genetically diverse background.

## Discussion

Murine primary subcutaneous adipocytes provide a valuable model for studying beige fat biology, facilitating research on cell-autonomous beige adipogenesis, thermogenic mechanisms, and functional roles of beige adipocytes. This system also serves as a platform for screening small molecules that stimulate beige fat thermogenic activation and recruitment. Key advantages include flexibility in experimental design and the ability to make direct comparisons across models under identical culture conditions. These cells closely mimic the native adipocyte phenotype of specific subcutaneous depots and retain the genetic and epigenetic characteristics of the donor organism, features often lost in immortalized cell lines. While primary mature adipocytes offer the highest in vivo relevance, their fragility and buoyancy limit their use in culture-based assays, making differentiated primary adipocytes a more practical and versatile alternative.

However, culturing and differentiating primary preadipocytes present several challenges. These include variability introduced by the enzyme digestion-based SVF isolation process, which can impact preadipocyte viability and surface marker expression, the heterogeneity of the SVF population, and the limited proliferative capacity of preadipocytes. Additionally, primary preadipocytes are highly sensitive to culture conditions, and their differentiation capacity can vary significantly even under optimized protocols due to factors such as the precise composition of the culture medium and individual mouse characteristics (e.g., sex, age) [50]. Consequently, these variables can impact experimental outcomes.

Among these challenges, the induction and differentiation of preadipocytes remain the most critical steps in this system. In this study, we conducted an unbiased evaluation of adipogenic cocktails currently used for the induction and differentiation of primary mouse inguinal preadipocytes. Our findings demonstrate that the composition of adipogenic inducers significantly affects both adipogenic and thermogenic efficacy. Rosi emerged as a key component during the differentiation phase for maximizing beige adipocyte biogenesis. However, the resulting mature adipocyte cultures had limitations in fully representing thermogenic activation at the transcriptional level in response to external stimuli, such as β-AR agonists, while acute signal transduction and real-time functional responses remained strong.

For screenings to identify compounds that enhance beige adipocyte activation or biogenesis at the transcriptional level, primary inguinal preadipocytes could be differentiated in adipogenic medium with limited Rosi exposure to avoid masking thermogenic activation by saturated basal activity. Among the adipogenic inducers used, T3 and indomethacin may be dispensable for promoting beige adipogenesis in primary mouse inguinal preadipocytes. Furthermore, selecting suitable FBS containing essential growth factors and hormones is critical for achieving effective differentiation.

The most commonly used cell lines for thermogenic research are 3T3-L1 and C3H/10T1/2 cells, both established from murine embryos. Over the years, adipocyte differentiation protocols for these cells have been well refined. The 3T3-L1 cell line is the most widely used preadipocyte model for studying adipocyte development and biology [51]. It has enabled breakthrough advances in understanding adipogenesis by facilitating the identification of pro-adipogenic compounds, such as insulin, IBMX, dexamethasone, and PPARγ agonists, as well as key transcription factors, including C/EBPβ and PPARγ [52]. Although differentiated 3T3-L1 preadipocytes express thermogenic adipose markers and exhibit increased mitochondrial respiration in response to catecholamines and various compounds [53–55], it should be noted that even upon stimulation, UCP1 expression remains low [56]. Therefore, 3T3-L1 cells may not be considered a bona fide thermogenic adipose model. In contrast, murine mesenchymal multipotent C3H/10T1/2 cells have been particularly useful for investigating beige adipocyte thermogenic activity. Notably, PPARγ agonists have been shown to be essential during the differentiation phase for driving the adipogenic conversion of C3H/10T1/2 cells [51].

Previous studies have demonstrated that Rosi promotes beige thermogenesis across multiple cell culture models, including murine 3T3-L1 and C3H/10T1/2 cell lines, as well as primary mouse and human subcutaneous adipose SVF cultures. These studies consistently reported increased thermogenic gene expression (e.g., *Ucp1*), enhanced mitochondrial biogenesis, increased glucose uptake, and/or elevated oxygen consumption [19, 36, 57, 58]. Many studies used Rosi throughout the entire adipogenic process, whereas others restricted exposure to the early stages (induction and the first few days of differentiation) or applied it to fully differentiated mature adipocytes to activate thermogenesis. Notably, Ohno et al. showed that chronic Rosi exposure (≥3 days) is necessary for effective browning [36]. In their study, when Rosi was limited to 2 days of induction and the first 2 days of differentiation within an 8-day adipogenic timeline (i.e., 4-day treatment followed by 4-day withdrawal), activation of thermogenic molecular programs was not sustained or robustly induced [36]. These findings suggest that Rosi exerts a critical effect during the differentiation phase or that continuous exposure throughout adipogenesis is required to achieve maximal thermogenic activation—consistent with our findings.

As a PPARγ agonist, Rosi binds to and activates PPARγ, enhancing transcription at PPAR response elements in the promoters or enhancers of thermogenic genes [59, 60]. Although the precise mechanisms underlying Rosi-induced beige thermogenesis are not fully defined, evidence indicates that SIRT1-dependent deacetylation of PPARγ facilitates PRDM16 recruitment and that post-translational pathways can stabilize PRDM16 [36, 41, 61]. However, these do not fully account for Rosi-induced beiging, as beige adipocytes can arise without PRDM16, including in our study, and additional pathways such as NRF2-mediated inhibition of autophagy have been reported [36, 62, 63]. TZDs, including Rosi, have also been suggested to influence mesenchymal stem cell lineage allocation toward adipogenesis; however, subsequent work indicates that TZDs enhance adipogenesis without directly altering lineage commitment [64, 65]. Overall, our findings are consistent with Rosi primarily augmenting beige adipogenesis rather than determining lineage commitment, as induction-phase–limited exposure yielded modest recruitment of thermogenic adipocytes.

In conclusion, differentiating beige adipocytes from mouse subcutaneous adipose SVF cells using a conventional culture system remains one of the most popular and practical approaches, balancing technical feasibility with physiological relevance. However, variability in beige adipogenesis and the characteristics of beige adipocytes, determined by different combinations of adipogenic inducers, highlight the importance of carefully selecting or optimizing these cocktails to align with specific experimental objectives and accurately represent beige adipose biology and functional responses.

## Supplementary information

Supplementary materials

## Data Availability

The datasets used during the current study are available from the corresponding author on reasonable request.
