# Supplementary material for: Optimizing adipogenic cocktail composition to enhance beige adipogenesis and evaluate thermogenic potential in primary mouse subcutaneous fat cell cultures
Source: Int J Obes (Lond). 2025 Nov 24;50(2):386–96. doi: 10.1038/s41366-025-01946-8 (PMC12912031; doi:10.1038/s41366-025-01946-8)
Supplement: Supplementary file 1 — Supplementary materials [file 41366_2025_1946_MOESM1_ESM.docx]

**Ma et al.**

**Supplementary Table 1**

**Table S1.** Adipogenic inducers used in adipogenic cocktails.

| **Chemicals** | **Stock concentration** | **Solvent** |
| --- | --- | --- |
| IBMX | 0.5 M | DMSO |
| Insulin | 850 µM | 0.1N HCl |
| T3 | 10 µM | 1 N NaOH |
| Indomethacin | 1.25 mM | 1 M HCl |
| Rosi | 10 mM | DMSO |

IBMX, 3-isobutyl-1-methylxanthine; Rosi, rosiglitazone; T3, triiodothyronine

**Ma et al.**

**Supplementary Table 2**

**Table S2.** Primer sequences.

| **Gene** | **Forward Primer** | **Reverse Primer** |
| --- | --- | --- |
| *Tbp* | GAAGCTGCGGTACAATTCCAG | CCCCTTGTACCCTTCACCAAT |
| *Ucp1* | GGCATTCAGAGGCAAATCAGCT | CAATGAACACTGCCACACCTC |
| *Pparg* | GGAAGACCACTCGCATTCCTT | GTAATCAGCAACCATTGGGTCA |
| *Adipoq* | AGATGGCACTCCTGGAGAGAAG | ACATAAGCGGCTTCTCCAGGCT |
| *Fabp4* | TGAAATCACCGCAGACGACAGG | GCTTGTCACCATCTCGTTTTCTC |
| *Cox8b* | CCGAGAATCATGCCAAGGCT | CCTGCTCCACGGCGGAA |
| *Ppargc1a* | GAATCAAGCCACTACAGACACCG | CATCCCTCTTGAGCCTTTCGTG |

**Ma et al.**

**Supplementary Figure 1**


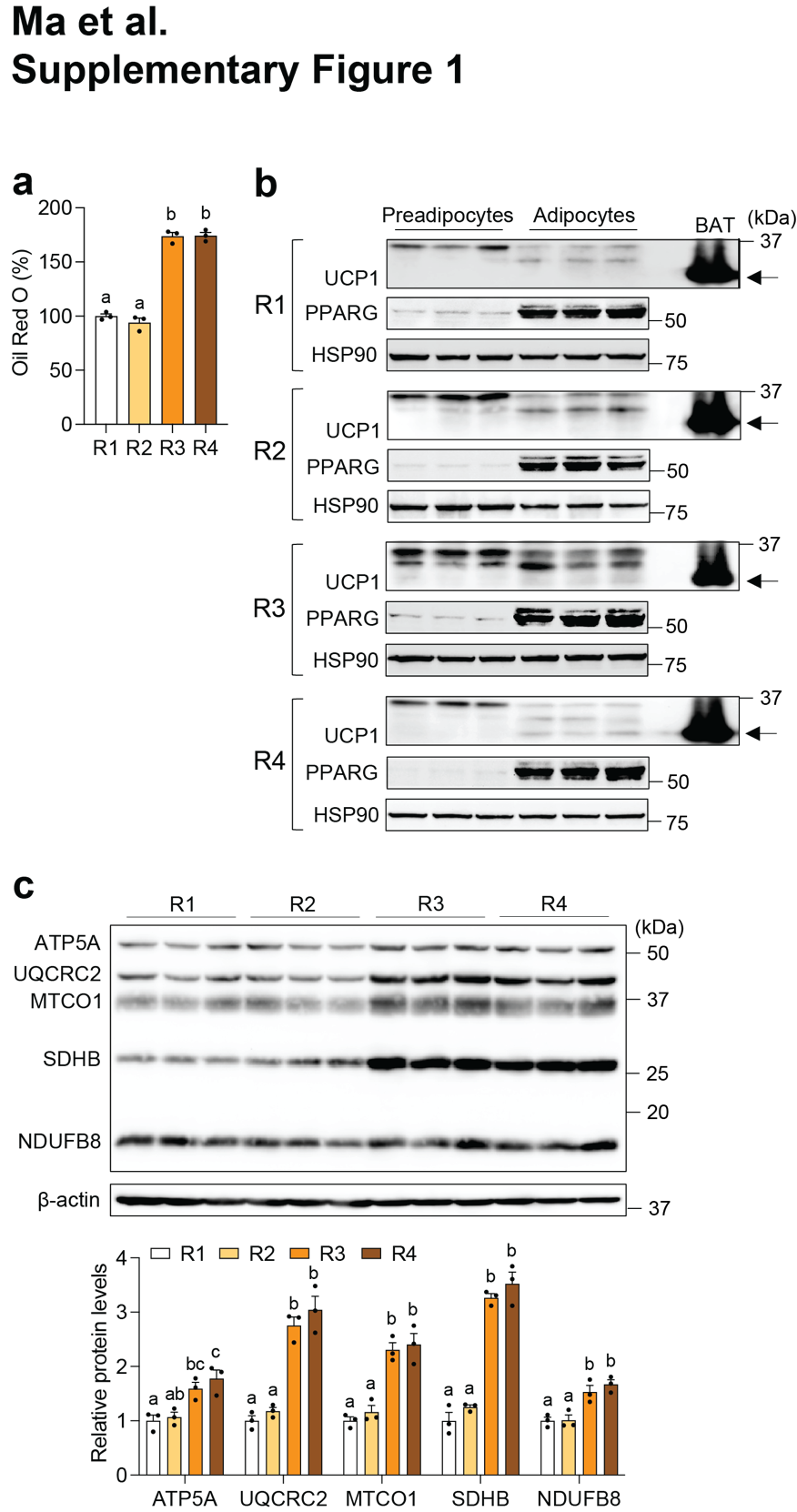


**Figure S1. The effects of different adipogenic recipes on beige adipogenesis in primary inguinal preadipocytes from wild-type C57BL/6J mice.** (**a**) Quantification of neutral lipids stained with Oil Red O in mature adipocytes differentiated with the four adipogenic cocktails (n=3/group). (**b**) Immunoblot analysis of UCP1 and PPARG in preadipocytes and mature adipocytes differentiated with the four adipogenic recipes, with HSP90 as a loading control (n=3/group). Brown adipose tissue (BAT) was used as a positive control for UCP1, indicated by the arrow. (**c**) Immunoblot analysis of mitochondrial respiratory chain complex proteins in mature adipocytes differentiated with the four adipogenic recipes, with β-actin as a loading control. ATP5A (Complex V, ATP synthase), UQCRC2 (Complex III, cytochrome bc1 complex), MTCO1 (Complex IV, cytochrome c oxidase), SDHB (Complex II, succinate dehydrogenase), and NDUFB8 (Complex I, NADH:ubiquinone oxidoreductase). Protein levels were quantified by densitometry and normalized to HSP90 (n=3/group). Data are presented as mean ± SEM. Four-group comparisons were assessed by one-way ANOVA with Tukey’s post hoc test. Different letters indicate statistically significant differences at *p* < 0.05.

**Ma et al.**

**Supplementary Figure 2**


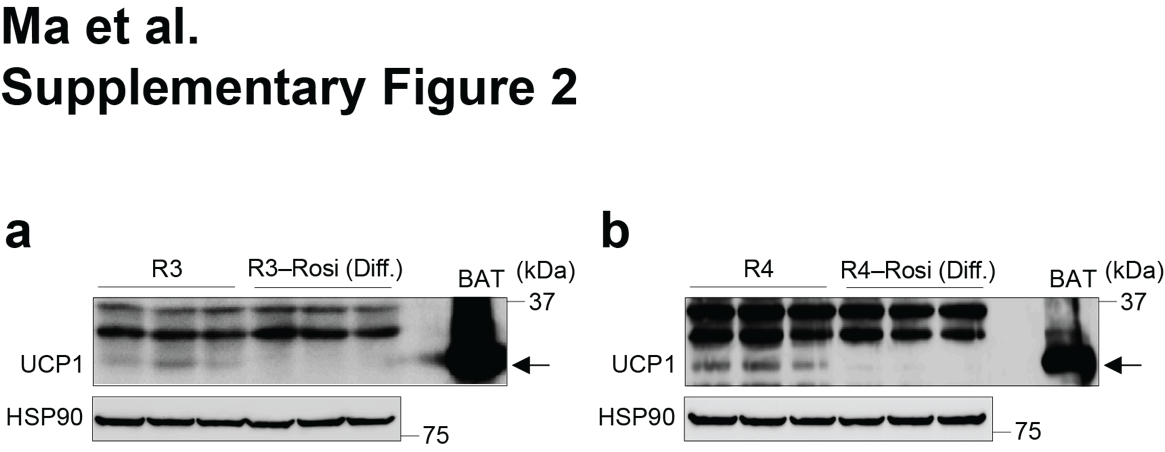


**Figure S2. The effects of rosiglitazone (Rosi) in the differentiation phase of beige adipogenesis in primary inguinal preadipocytes from wild-type C57BL/6J mice.** (**a, b**) Immunoblot analysis of UCP1 in mature adipocytes differentiated using adipogenic recipes (**a**) R3 and (**b**) R4 with Rosi either retained or removed during the differentiation phase (n=3/group). HSP90 was used as a loading control. Brown adipose tissue (BAT) served as a positive control for UCP1, indicated by the arrow.

**Ma et al.**

**Supplementary Figure 3**

**
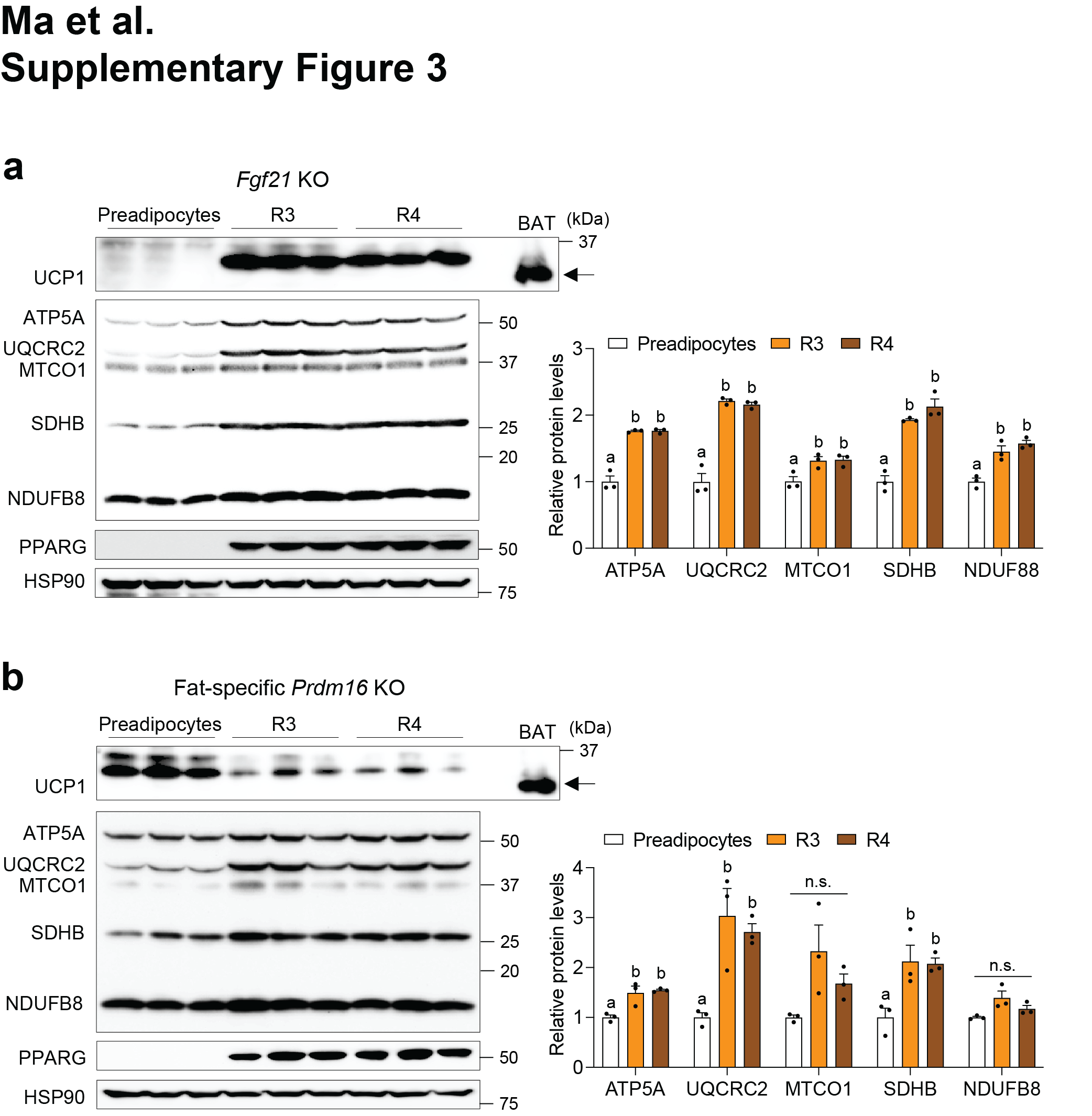
**

**Figure S3. Beige adipogenesis in primary inguinal preadipocytes of genetically modified mice.** (**a, b**) Immunoblot analysis of UCP1 and mitochondrial respiratory chain complex proteins in preadipocytes isolated from (**a**) *Fgf21* knockout (KO) and (**b**) fat-specific *Prdm16* KO mice, and in their mature adipocytes differentiated with adipogenic recipes R3 and R4. PPARG and HSP90 served as the adipogenic and loading controls, respectively. Brown adipose tissue (BAT) was included as a positive control for UCP1, indicated by the arrow. Mitochondrial proteins analyzed: ATP5A (Complex V, ATP synthase), UQCRC2 (Complex III, cytochrome bc1 complex), MTCO1 (Complex IV, cytochrome c oxidase), SDHB (Complex II, succinate dehydrogenase), and NDUFB8 (Complex I, NADH:ubiquinone oxidoreductase). Protein levels were quantified by densitometry and normalized to HSP90 (n=3/group). Data are presented as mean ± SEM. Four-group comparisons were assessed by one-way ANOVA with Tukey’s post hoc test. Different letters indicate statistically significant differences at *p* < 0.05; n.s., not significant.
